# Looking Back to Move Forward: How Review Articles Could Boost Forensic Entomology

**DOI:** 10.3390/insects12070648

**Published:** 2021-07-15

**Authors:** Damien Charabidze, Daniel Martín-Vega

**Affiliations:** 1 UMR 8025, Centre d′Histoire Judiciaire, Université de Lille, F-59000 Lille, France; 2 Unit of Social Ecology (USE), Université Libre de Bruxelles (ULB), 1050 Bruxelles, Belgium; 3 Departamento de Ciencias de la Vida, Universidad de Alcalá, 28805 Alcalá de Henares, Madrid, Spain

The Locard′s exchange principle (1930) holds that the perpetrator of a crime leaves traces behind that can later be sampled and used as forensic evidence. On the contrary, insects are autonomous cues: they appear, grow and move away from a crime scene without any human action. Forensic entomologists rely on these living cues to reconstruct a posteriori the post-mortem chronology.

In the Editorial of the first Forensic Entomology Special Issue (2001), Benecke [1] stated “*There are three things that we need in forensic entomology: more young researchers (…), open discussion about possible flaws in our methods and PMI calculations, and access to information for scientists all over the world (…)*”. Thanks to research, forensic entomology has since investigated several possible flaws and evolved from a rather empirical clue into a mature evidence-based forensic science. This is the consequence of the deep involvement of many students and researchers worldwide, who performed extensive studies and published hundreds of articles, case reports and technical refinements. However, this may be too much: the above-mentioned easy access to information has on the way been blurred by an excessive amount of publication. Revealingly, the current top 3 cited “forensic entomology” documents are rather old manuals from 1992, 1991 and 2007 and many forensic pathologists and crime scene investigators still think in terms of “squads”, a hypothesis yet abandoned by forensic entomologists for decades. Thus, while several factors may explain the slow rise of forensic entomology, its complexity and inability to simply answer some practical questions cannot be ignored. In this context, we believe review articles may be the next big step for forensic entomology.

Although their main (and obvious) disadvantage is that they will eventually become outdated, review articles integrate and synthesize relevant information that otherwise would be dispersed, so they can be both the perfect introduction to a novel topic [2] and a new starting point that boosts research within a particular area of knowledge. Either if they are written from a purely “narrative” perspective or with a systematic and/or meta-analytical approach, review articles are potentially useful tools for forensic entomology practitioners, researchers and students. Narrative reviews can find their place either in academic journals or in academic collaborative books (e.g., [3,4]), whereas systematic reviews that are developed around a research question and often include a meta-analysis of published data are more often published among other scientific papers in academic journals.

*“Oh my god. You still think forensic entomology is all blowflies and screw-worms, don’t you?”*

Having trouble finding trace amounts of blood at a crime scene, police inspectors of the TV show *Brooklyn 99* call a superstar forensic entomologist, Dr. Yee (Season 6, Episode 10, March 2019). Supposedly the scientist has bred a species of flies that can detect small traces of blood, even when it has been cleaned. Unfortunately, Dr. Yee was a fraud. But while forensic entomology is still about blowflies and maggots, and not mutant flies, some practices of forensic entomology have greatly evolved during the last 40 years. In the first article of this Special Issue, UK forensic entomologist M. Hall shares his long experience and his point of view on the links between fundamental research and its application in forensic cases [5]. Review articles are one of these bridges, as they are useful for all, can focus on any step of the forensic entomology analyses and can be written by both academics and case-workers (contrary to books that require long-time and deep knowledge). Unfortunately, such valuable sources of knowledge currently seem under-represented compared to classical research (Figure 1).

Due to their low number, forensic entomologists cannot move to all crime scenes, and sampling is thus often performed by non-specialists [6]. Subsequent transport, conservation and temperature control are also extremely important for forensic entomology analysis. To avoid bias, crime scene technicians or forensic pathologists who sample insects must follow clear, concise and efficient sampling, fixation and conservation protocols. While the *Best practice in forensic entomology—standards and guidelines* paper published by Amendt et al. in 2007 [7] remains one of the most cited papers in forensic entomology, it provides general guidelines rather than a clear sampling protocol and is now out of date. In practice, each country or even laboratory currently has its own procedures, and there is a lack of widely accepted international standards. Furthermore, researchers are still working on some key points. As an example, up-to-date protocols are still needed for those eventual cases where eggs could be the only entomological evidence recovered in a death scene. While Amendt et al. [7] recommended to kill and preserve egg specimens—if maintaining living samples was not possible—by directly placing them in 70–95% ethanol, it was recently shown that this method results in a marked decomposition of tissues, thus making it impossible to reliably use age-specific morphological markers [8]. Similarly, the fixation of fly larvae using very hot water prior to storage in 70–95% ethanol requires anticipation and material and is most of the time difficult or impossible under harsh crime scene conditions. Finally, the guidelines by Amendt et al. [7] did not provide any recommendations on how to fix and preserve the intra-puparial stages. However, this period can be particularly crucial in minimum post-mortem interval estimations as it can account for more than half of the developmental duration of the blow fly life cycle. Accordingly, in the last decade, a number of studies have tested the suitability of different fixation and preservation methods: at least eight different articles on sample storage have been published in the last ten years, including one in this Special Issue [9,10]. In this context, it is obviously difficult for a non-specialist to stay informed and make the right methodological choices. There is no doubt that an up-to-date review published under a large scientific consensus and establishing a step-by-step sampling protocol would be a great milestone for the whole forensic entomology community.

The next step in the forensic entomology process, species identification, is also crucial. While the adults of the most common necrophagous species can be easily identified, this task is more challenging for some incidental species or early instars. Whereas identification keys to both the larval and adult stages of the most common blow fly species occurring on cadavers have been published, only spare morphological descriptions might be available for other forensically relevant taxa, with the immature stages of many species remaining unknown. Molecular biology, and especially DNA-based methods, are thus widely investigated. In this Special Issue, Gzywacz et al. [11] provide a good example of the value of molecular methods when morphological identification is not possible. However, while powerful and increasingly accessible, these methods remain more expensive and time-consuming than traditional morphological identification. On the other hand, the idea of traditional morphological identification being a simple and straightforward method can be misleading. First, it requires a solid background in insect taxonomy and many forensic practitioners might not be properly trained in the observation of certain diagnostic characters. Furthermore, some diagnostic morphological characters may be obscured or altered if the sample preservation is not optimal or if the practitioner lacks expertise in the handling, processing and/or mounting of insect specimens. In this Special Issue, Pradelli et al. [12] present simple methods to clean puparial samples in order to facilitate their morphological identification. Some practitioners may also “feel tempted” to use keys from a different biogeographic region, sometimes leading to misidentifications. Thus, reviews focusing on simple and efficient DNA-based methods and straightforward taxonomic keys dedicated to carrion insects, clearly delimited to particular biogeographical regions and supported by unambiguous, high-resolution images of the most useful diagnostic characters, should be encouraged.

Beside molecular species identification, significant changes occurred in forensic entomology during the last decades. In the early 1990s, the “squads” method, first formalized by J.P. Megnin a century ago [13], was progressively abandoned. As summarized in the Crime Scene Investigation TV show, this idea was simple and appealing: “*Insects arrive at a corpse in a specific order. Like summer follows the spring. And you can pinpoint time of death, based on the type and age of insects present on the body*” (Grissom, CSI Las Vegas 1.10). However, what happens in the field is rarely simple enough to be summarized in a punchline, and Megnin’s necrophagous squads were not reliable enough for forensic purposes [14]. Research indeed evidenced that using the chronological faunal succession for post-mortem interval estimations actually requires large-scale local studies involving numerous cadavers, repetitions over years and extensive statistical analysis [15]. In this Special Issue, LeBlanc et al. [16] show how the occurrence and assemblage composition of necrophagous flies in small bait traps may differ from that of cadavers, thus warning about the potential issues of extrapolating trap collection data to courtroom proceedings. Thus, the step between field experiments and forensic cases application is high: Moreau [15] reviewed these pitfalls, but also acknowledged the interest of ecological decomposition experiments. He suggest that “the authors should explicitly recognize that the (successional field) study is descriptive, thus not allowing for transposition of the results to other situations or use in court”.

On the contrary, development–time datasets, which are quite simple to perform and an indispensable prerequisite for aging larvae, have received little attention during the last years. In this Special Issue, Matuszewski [17] highlights the need for development datasets for potentially useful species that regularly breed on cadavers, but for which no reference developmental data are available. For those species with wide geographical distributions, local developmental studies are required as there might be differences in the developmental rates between populations. In this context, Shin et al. [18] present baseline developmental data for the blow fly species *Lucilia sericata* in South Korea, commonly used as a forensic indicator and for which development–time datasets from other geographical regions are available [17]. Regrettably, many researchers may be discouraged to perform basic developmental studies as they could sometimes be seen as “not innovative enough” for being published in some high-profile academic journals. In addition, as in the case of sample collection at the forensic scene, an updated review providing detailed and standardized rearing and sampling protocols for developmental studies would be highly beneficial and enable the comparison of different datasets [17]. Laboratory analyses have indeed demonstrated that numerous biotic and abiotic parameters such as food type, larval behavior or bacterial load, to name a few, could significantly affect larval development [19]. However, most of these studies lack concrete applied methodologies to take account of the effect observed experimentally. In such a situation, forensics readers may wonder how they are supposed to proceed, and the most pessimistic observers would question the reliability of forensic entomology analyses. Thus, while there is currently almost no review dedicated to synthesizing the biology and development of key species (e.g., *Lucilia sericata*), such reviews could distinguish incidental effects from clear trends and suggest appropriate case-related methodology.

Even if there are some key areas where much research still needs to be carried out [17], forensic entomology is today a dynamic science, with the above-mentioned increasing number of scientific publications and active researchers and practitioners across the world. However, many other researchers and practitioners can face significant challenges if they approach this discipline for the first time. Again, updated and comprehensive review articles can be a valuable tool for those who aim to start forensic entomology in a particular country or region. In this Special Issue, Wang et al. [20] review the recent progress in forensic entomology research and application made in China, providing an inventory of the different types of baseline studies conducted, as well as highlighting the need for proper entomological training for forensic practitioners.

Collaboration between forensic practitioners and researchers is indeed of mutual benefit as it enables access to large forensic cases databases, providing new insights into the biology, ecology and forensic relevance of the insect species associated with human cadavers. Such meta-analyses of forensic cases can identify, for example, patterns in the seasonal activity, the colonization of cadavers or the co-occurrence with other species, thus improving the interpretation of further forensic entomological evidence and stimulating additional research on forensically relevant species (e.g., [21,22]). They can also shed some light on the forensic implications of traditionally neglected aspects of the insect activity on cadavers (e.g., [23]). However, these types of *a posteriori* analyses of forensic databases are still scarce. More frequent is the publication of case reports addressing one or a few particular cases (e.g., [24]). The publication of those particular case reports can be useful if, for example, they describe the occurrence of a species which had not previously been considered as forensically relevant [25], highlight how an entomological analysis can provide new and significant insights into a case [26] or show other utilities and applications of the evidence [27]. Nonetheless, meta-analyses of large forensic cases datasets are desirable as they provide strong reference data for the most usual and required type of entomological analyses in case reports. In this sense, the preparation of a solid case report is pivotal, as it must show the reliability and significance of the performed entomological analyses. In this Special Issue, Kotzé et al. [28] propose an overview of sections to be considered when drafting a forensic entomology case report, which could certainly contribute to the standardization of these reports among both experienced and novel practitioners.

As a very specific area of study, forensic entomology is more often requested in unusual cases that require additional and/or alternative approaches, rather than routinely applied in forensic casework. Even if a case requiring an entomological analysis is not particularly “unusual”, it will always be unique, differing spatially and temporally from a priori similar cases [5]. Therefore, the conclusions drawn from that entomological analysis and stated in a forensic report will be opinions based on science rather than undisputable scientific facts. That those opinions should be based on science means that they should rely both on a solid experience in the area and on related and published peer-reviewed studies. This emphasizes the importance of forensic entomology research, either if it consists of baseline studies stimulated by general needs (e.g., development–time datasets) or on ad hoc experiments simulating the conditions of a specific case [5,17]. On the other hand, this also highlights the need for self-criticism and awareness among researchers and practitioners of the limitations of the application of forensic entomology [29,30].

As pointed out by Hall [5] in the opening of this Special Issue, *“Opinion evidence is clearly important, but the opinions expressed in an expert witness statement come from an interpretation of the facts of the case (e.g., the presence of larvae or the temperatures recorded) in the light of the expert witness’ experience and their knowledge of data (e.g., rates of insect development) that are generated through research focused on supporting casework”*. Accordingly, this Special Issue presents a series of remarkable research studies, forensic case reports and reviews, all of them highlighting the complex and challenging implications of applying forensic entomology studies to court. It is our opinion that it might be the right time for critical, disruptive reviews that clearly identify the weaknesses and limitations of our field. Acknowledging the real nature of forensic entomology and its inherent or current limitations will avoid misinterpretations, overstatements and troubles in court. It may be the basis for a more probabilistic/Bayesian approach to the forensic entomology analysis, transforming what might appear to be weaknesses into a strength.

## Figures and Tables

**Figure 1:**
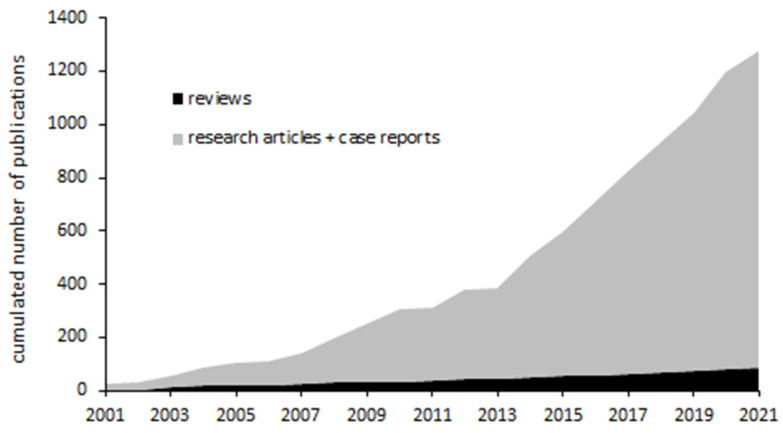
Cumulated number of reviews (black) and research (grey) articles published during the last 20 years (PubMed research performed in March 2021 using “forensic entomology” as keyword).
